# High Enantioselectivity in Adsorption of Chiral Molecules on the Surface of Chiral Terbium Phosphate Nanocrystals

**DOI:** 10.1021/jacs.4c16883

**Published:** 2025-04-18

**Authors:** Abdullah Idrees, Bar Reuven, Gil Markovich

**Affiliations:** School of Chemistry, Tel Aviv University, Tel Aviv 6997801, Israel

## Abstract

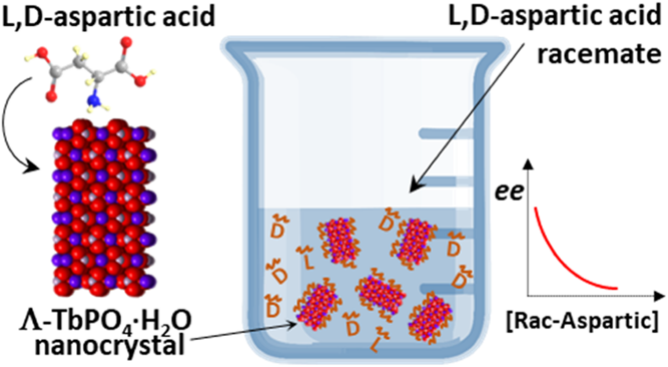

The enantioselective adsorption (and catalysis) of molecules on chiral surfaces has been a topic of interest, in particular with respect to models describing homochirality in the evolution of life. While such adsorption on single crystal surfaces has been studied using various traditional surface science techniques, similar studies on powders have been less successful. Here we show that studying enantioselective adsorption on the particular system of intrinsically chiral TbPO_4_·H_2_O nanocrystals (chiral space group) is highly useful and obeys some simple rules. The advantages of this system are the ability to produce such nanocrystals with 100% enantiomeric purity with virtually no ligands, due to strong autocatalytic effects in their enantioselective nucleation, and the prevalence of one type of surface type (100) in the formed nanocrystals. We show that particular chiral molecules such as tartaric and aspartic acids exhibit significant enantioselectivity in adsorption, while similar ones, like glutamic acid, show none. We demonstrate that at least three functional groups are required to define enantioselective adsorption, and in addition, there should be matching of distances between these groups and the corresponding surface adsorption sites (Tb^3+^ sites in our case).

## Introduction

The study of chiral molecular surface chemistry is critical for advancing enantioselective heterogeneous catalysis, providing an alternative to standard homogeneously catalyzed methods for creating enantiopure molecules.^1−3^ Chiral separation in chromatography is another important use, where chiral stationary phase design requires a thorough understanding of the interactions between chiral molecules and surfaces.^4−7^ Furthermore, it has been suggested that catalytic reactions on mineral surfaces in the primordial environment could have been the source of life’s homochirality.^8,9^ Chiral surface preparation can be accomplished in a few different ways: By templating chiral molecules onto achiral surfaces,^10^ or due to their inherent atomic structure.^11^ High-Miller-index naturally chiral surfaces (of achiral crystals) lack mirror symmetry because they reveal asymmetric terrace, step, and kink features.^12^ The adsorption energies,^13^ orientations, and aggregation of chiral adsorbates may all be impacted by the surface’s chirality.^14,15^ The kinds of enantioselective properties needed for enantioselective chemical processing may be present on these naturally chiral surfaces.^16,17^

Chiral bulk structures are observed in many crystalline substances, such as quartz and cinnabar, and of course, crystals of chiral organic molecules that are enantiomerically pure; the chirality of the bulk determines the chirality of the crystalline surfaces exposed by cleavage of these crystals.^18,19^ In recent years, the preparation of nanocrystals (NCs) of chiral inorganic materials with high enantiomeric excess (*ee*) has been reported,^20,21^ and in particular, the synthesis of TbPO_4_·H_2_O NCs (space groups *P*3_1_21/*P*3_2_21) was shown to achieve 100% *ee* using a chiral ligand such as tartaric acid (TA).^22−24^

**Scheme 1:**
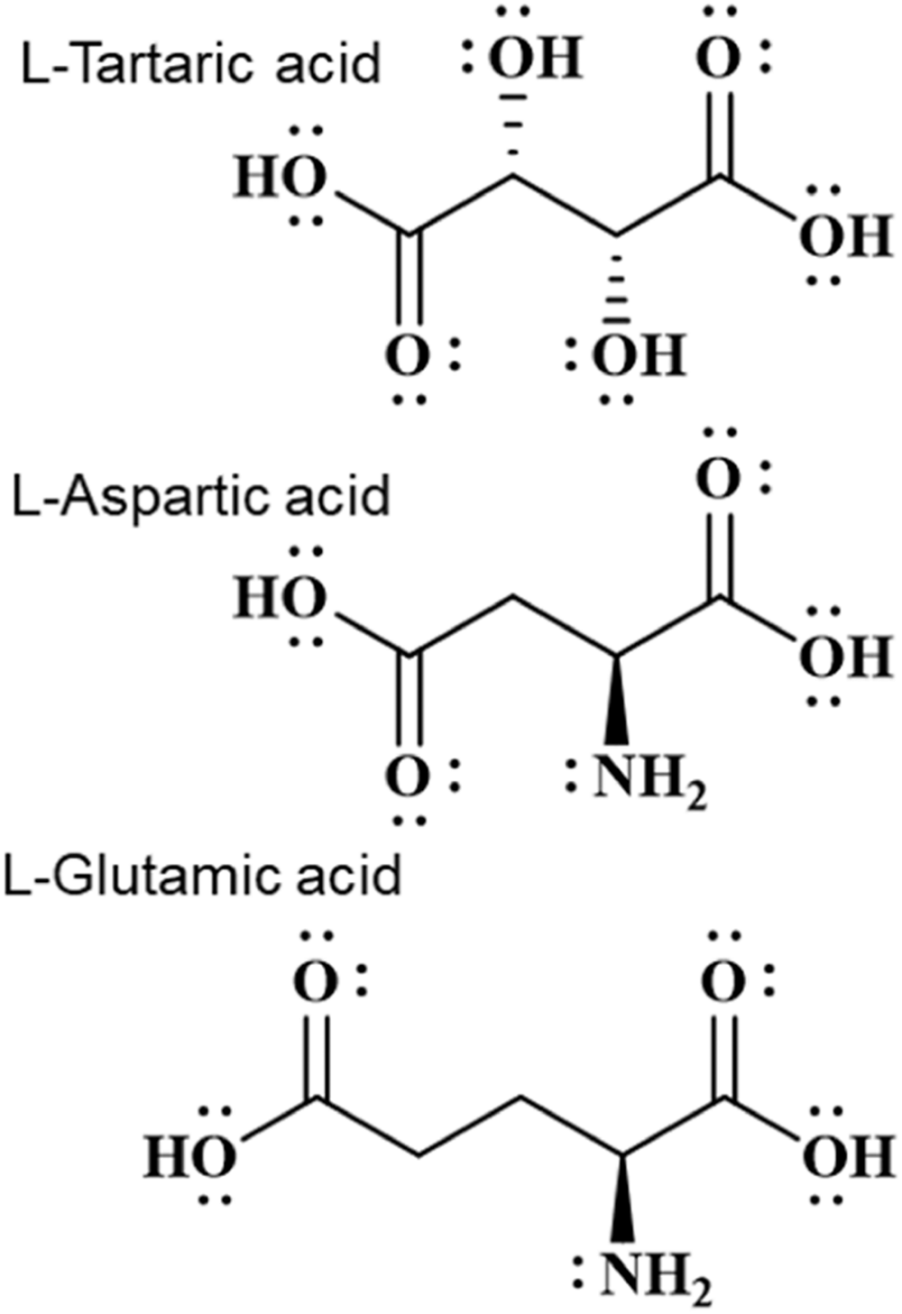
Molecules Studied in the Chiral Adsorption Experiments

Here we report on enantioselective adsorption on the naturally chiral (low Miller index) surface of TbPO_4_·H_2_O NCs. We examined the interactions between the chiral surfaces of TbPO_4_·H_2_O NCs and chiral biomolecules, including aspartic acid (ASP), glutamic acid, and TA-containing carboxyl, hydroxyl and amine functional groups bound to have strong coordinative interaction with the Tb^3+^ ions (see Scheme 1). Circular dichroism (CD) spectroscopy was employed to investigate the enantioselective adsorption of the chiral molecules. This study involves adsorbing both pure enantiomers and racemic mixtures of TA or ASP to the chiral surface.

## Experimental Section

### Enantiopure TbPO_4_·H_2_O NC Synthesis Procedure

Terbium chloride hexahydrate (99.9%) was purchased from Fisher Scientific. Sodium dibasic phosphate (≥99.0%), d-TA acid (99%), l-TA acid (≥99.5%), racemic dl-TA (99%), l-ASP (≥99%), d-ASP(≥99%), dl-glutamic acid (≥99.0%), and dl-methyl succinic acid (99%) were purchased from Sigma-Aldrich. Hydrogen chloride (32%) was purchased from BioLab. All chemicals were used without further purification. All water used was ultrapure (18.2 MΩ), obtained from a Millipore Direct-Q3 UV system. The racemate of ASP was prepared by mixing the two enantiomers in equal volumes and slightly correcting the quantity of one of the enantiomer solutions to obtain a zero CD signal.

The NC seed particles were synthesized enantioselectively following a procedure of Schwartz et al.^24^ To 10 mL of water, we added 285 μL of 100 mM Terbium chloride solution at acidic pH (pH∼ 2) heated to 50 °C, followed by the rapid addition of 600 μL of 100 mM Na_2_HPO_4_ solution, also heated to 50 °C, to the terbium chloride solution under stirring. The solution was stirred for 1 h at 50 °C, attaining a milky appearance. The obtained enantio-pure NCs were labeled Λ-NCs if prepared with l-Tartaric acid (l-TA), and Δ-NCs if prepared with d-Tartaric acid (d-TA). For seeding experiments, 100 μL of the NC solution out of the total ∼11 mL synthesized, which is ∼1% of the total synthesis quantity was taken. Enantio-pure NCs without ligands were prepared by repeating the same procedure as the above synthesis, only without any TA, but with the addition of the enantiopure Λ-NCs or Δ-NCs. The NCs were purified by centrifugation. Considering that the added seed particle solution contained ∼1% of the original TA quantity, their concentration at the “ligand-less” synthesis was about 0.1 mM. After the final purification of the resulting “ligand-less” NCs which removed practically all the dissolved residual TA, we estimate that <10% of the above concentration remains adsorbed to the NCs (see also Supporting Information, Table S6). Hence, a very small amount of TA, of equivalent concentration <10 μM, is left in the purified NC sample.

### Adsorption of Chiral Molecules onto the TbPO_4_·H_2_O NCs

The precipitate of purified NCs prepared by the seeded synthesis (after centrifugation at 3300 RCF for 10 min) was added to a 2 mL solution containing either pure l- or d-TA, l- or d-ASP, or a racemic mixture of rac-TA or rac-ASP, at various concentrations, and stirred for 2 h. The pH value of the solutions during adsorption was around ∼3, which is the natural pH of the aqueous solutions of the two acids at a few mM concentrations. Following the adsorption period, the NC samples underwent centrifugation at 3300 RCF for 10 min and the supernatant was separated for free enantiomer concentration analysis.

### Determination of the Total Adsorbed Equivalent Concentration of TA and ASP for Each Initial Concentration of the Acid Enantiomers

Initially, calibration curves for TA and ASP enantiomer concentrations using the UV CD peak heights at 215 and 204 nm, respectively, were plotted (Supporting Information, Figure S1). CD spectra were measured using an Applied Photophysics Chirascan CD spectrometer. The total adsorbed equivalent concentration of TA and ASP on the chiral surface of TbPO_4_·H_2_O NCs was determined for various total initial concentrations of the acids (Supporting Information, Figure S2 and Tables S2, S3). In the case of pure enantiomer adsorption, the calculation was simple:

Adsorbed equivalent concentration = initial enantiomer concentration – concentration of unadsorbed enantiomer estimated from the CD measurement.

### Analysis of Adsorption from a Racemic Mixture of TA and ASP

The CD measurement provided the difference between the concentrations of the two enantiomers in the free acid solution ([*L*]–[*D*], see Figures S3, S4 and Tables S4, S5), which was the opposite of the adsorbed equivalent concentration difference:
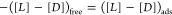


In order to determine the enantiomeric excess in each experiment, we need to know also the total adsorbed concentration of the two enantiomers ([*L*] + [*D*]). This was achieved by titrating the free acids remaining in the solution after separating the NCs with adsorbed molecules by centrifugation. NaOH was used as the titrant at concentrations of 0.4, 1.0, 2.0, and 3.0 mM for the different samples, to roughly match the order of magnitude of free acid concentration. The titration results were analyzed to quantify the amount of unadsorbed free acid concentrations, and by subtracting this from the initial acid concentration, the adsorbed equivalent concentration was calculated (see Figure S5 and Table S6). Furthermore, the *ee* was calculated using the following procedure:

From the titration curves:
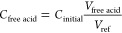
where *C*_free acid_ is the total concentration of the two unadsorbed enantiomers, *C*_initial_ is the initial racemate concentration before the adsorption experiment, *V*_free acid_ is the base volume required for titrating the two unadsorbed free acid enantiomers (determined from the titration curve’s derivative peak), and *V*_ref_ is the base volume required for titrating the initial racemate concentration.

Then the adsorbed equivalent concentration of both enantiomers was evaluated: *C*_ads_ = [*L*] + [*D*] = *C*_initial_–*C*_free acid_. Then, we could get the adsorbed *ee* estimate:
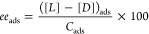


### Inductively Coupled Plasma Mass Spectrometry (ICP-MS) Analysis of the Solution in Contact with the NCs

Tb^3+^ and phosphate analysis were performed on NC samples purified after synthesis, comparing ultrapure water in contact with the NCs to 1 mM ASP solution incubated with the NCs. The two solutions were separated from the NCs by centrifugation and injected into an ICP-MS instrument (Agilent model 7800). The measurements were calibrated by Tb^3+^ and phosphate standard solutions.

## Results and Discussion

**Figure 1:**
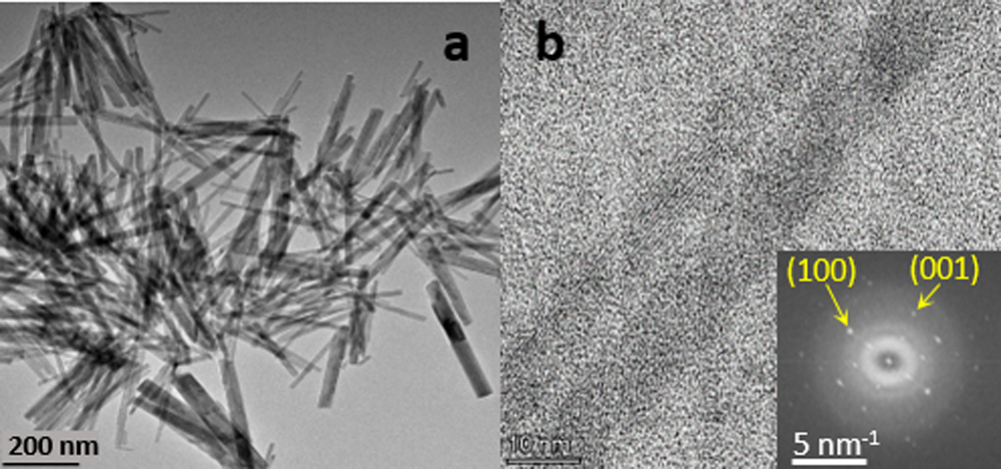
(a) Low-magnification TEM image of the synthesized TbPO_4_·H_2_O NCs. (b) High-magnification image of two NCs. Inset: FFT image of panel (b) with labeling of the spots corresponding to the *a* and *c-*axes.

The initial NC synthesis, performed at 50 °C, contained either l- or d-tartaric acid (TA), which has previously been shown to lead to the formation of enantiopure Λ- or Δ-NCs, respectively.^23^ Then, 1% of the total prepared NCs were used as seed particles for a new NC synthesis, without added TA, at 50 °C, which was previously shown to yield enantiopure NC samples, following the handedness of the seed NCs.^22,24^ After purifying this secondary NC sample by centrifugation, only traces of TA (<10 μM) were left at the NC sample. Figure 1a shows a TEM image of the final ligand-less NCs. Figure 1b presents a high-magnification TEM image revealing two single crystal nanorods. Fast Fourier transform (FFT) analysis of the HRTEM image shown in Figure 1b reveals the crystallographic planes present in the TbPO_4_·H_2_O NCs. Specifically, the FFT pattern indicates the presence of (100) type and (001) planes, consistent with the hexagonal phase structure of TbPO_4_·H_2_O NCs. This observation agrees with the X-ray diffraction results provided in Table S1 (Supporting Information). Analysis of many similar HRTEM images implies that all lateral facets of the NCs seem to correspond to the (100) type.

**Figure 2:**
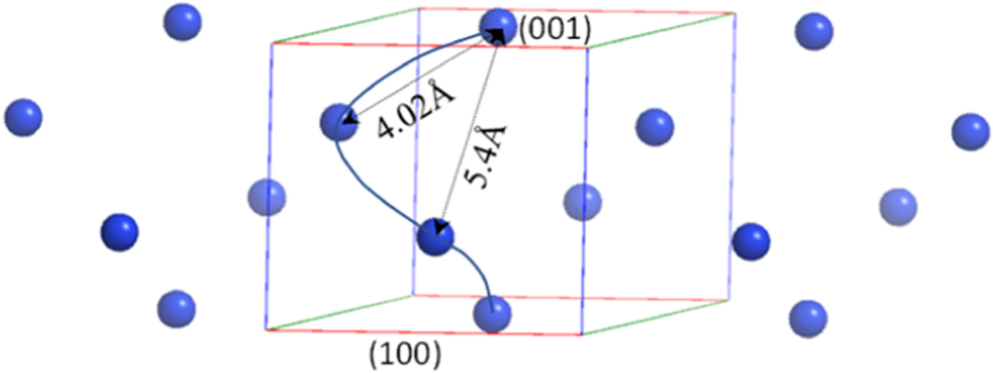
Distribution of Tb^3+^ ions at the (100) surface (side face) of the TbPO_4_·H_2_O crystal. The phosphate groups and water molecules were removed for clarity. The plotted frame defines the borders of a unit cell and the curved line connects the ions in one helix pitch encompassed by the unit cell, twisting along the *c*-axis (the screw axis). The nearest-neighbor and next-nearest-neighbor distances are indicated.

Hence, it seems that all the side facets of the NCs are of this single (100) family, consisting of about ∼95% of the surface area, and the minority face area, at the edges of the NCs is of the (001) type. This situation should be useful for achieving significant enantioselective adsorption of a suitable type of chiral molecules at the (100) family of facets, which are expected to have a chiral structure (see Figure 2). Hence, this chiral crystal system offers a potentially well-defined high-area chiral surface to perform enantioselective adsorption studies, unlike the case of quartz powders,^25^ for example, where many different facets of quartz are exposed, each with its different and sometimes opposite enantioselectivity.^8^

**Figure 3:**
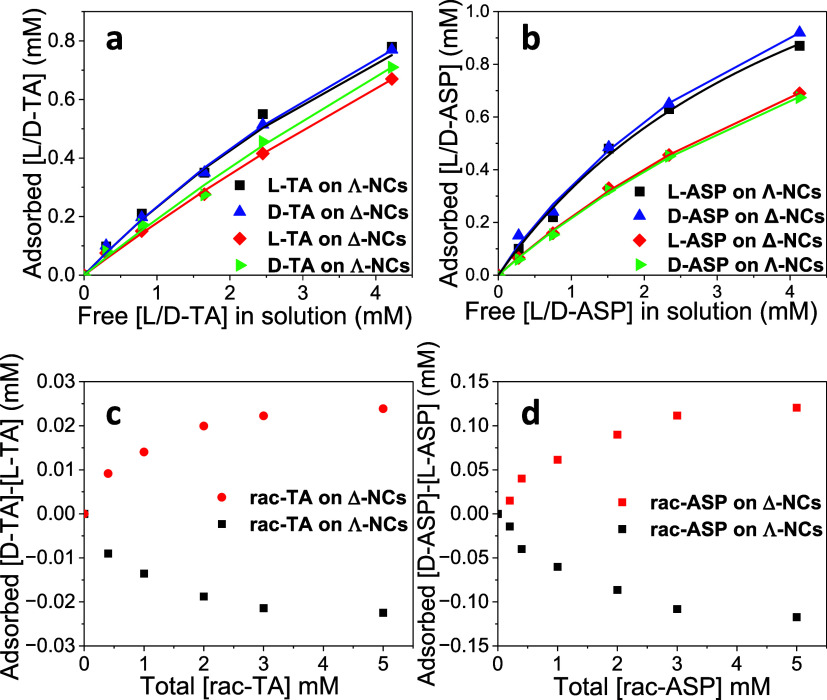
(a) Adsorbed amount of single TA enantiomers (in terms of equivalent adsorbed concentration) to constant amounts of Λ- and Δ-NCs as a function of free TA enantiomer concentration, where the symbols are the experimental data and lines are the fitted Langmuir model curves. (b) The same for ASP. (c) The difference in adsorbed enantiomer concentrations when a varying concentration of a racemic mixture of TA is brought in contact with a constant amount of Λ- and Δ-NCs. (d) The same for ASP. The zero points reflect CD measurements of the pure racemic mixture without interaction with the NCs.

The enantioselective adsorption of TA and ASP onto the chiral surface of the TbPO_4_·H_2_O NCs was investigated using CD spectroscopy. Calibration of TA and ASP enantiomer concentrations was done through CD measurements of enantiopure aqueous solutions of these molecules (Supporting Information Figure S1). Then, we have incubated the same amount of each type of NCs (Δ or Λ) with the same concentration of either pure l- or d-TA/ASP, separated out the NCs with the adsorbed molecules by centrifugation, and used CD measurement of the supernatant to establish how much of the added TA/ASP concentration was adsorbed to the NCs. As shown in Figure 3a,b, adsorbed TA/ASP equivalent concentrations varied with solution concentration. Notably, the adsorbed equivalent concentration of l-TA/ASP onto Λ-NCs and d-TA/ASP onto Δ-NCs exhibited significantly greater magnitudes compared to the adsorption of l-TA/ASP onto Δ-NCs and d-TA/ASP onto Λ-NCs. This difference, relatively large for the ASP case, underscores the preferential interactions between specific enantiomers of TA and ASP and the corresponding chiral NC surface.

We have also studied the enantiospecific adsorption of racemic TA and ASP molecular mixtures (rac-TA, rac-ASP) and the chiral surface of TbPO_4_·H_2_O NCs. CD measurements were conducted after the addition of rac-TA/ASP to solutions containing either Λ-NCs or Δ-NCs and the separation of the supernatant (free molecules). As rac-TA and rac-ASP lack net chirality, the CD data cannot reflect the adsorbed rac-TA/rac-ASP or specific enantiomer concentration on its own, as shown in Figure 3c,d, but a signal that is proportional to the *ee* in solution, which is opposite to the adsorbed one. The curves in Figure 3c,d demonstrate that adding rac-TA/rac-ASP to a solution containing either Δ-NCs or Λ-NCs leads to a CD signal which increases with total racemate concentration, suggesting a concentration-dependent adsorbed *ee*.

ASP demonstrated a significantly higher enantiomeric selectivity in its adsorption behavior than TA, both for the difference between single enantiomer adsorption and for the racemate. To estimate the enantiomeric excess in adsorption from the racemate, one would need to determine the total adsorbed equivalent concentration of TA and ASP for each initial concentration of the acids. This estimate was done by NaOH titration of the free acids left in solution after separation of the NCs with adsorbed molecules by centrifugation (see Experimental Methods and Supporting Information, Figure S5, and Table S6). Then, the difference in adsorbed equivalent concentration of the enantiomers shown in Figure 3c,d, was divided by the estimate of the total adsorbed equivalent concentration of acid molecules, which is the difference between the initial total concentration and the estimated free acid concentration from the titration.

**Figure 4:**
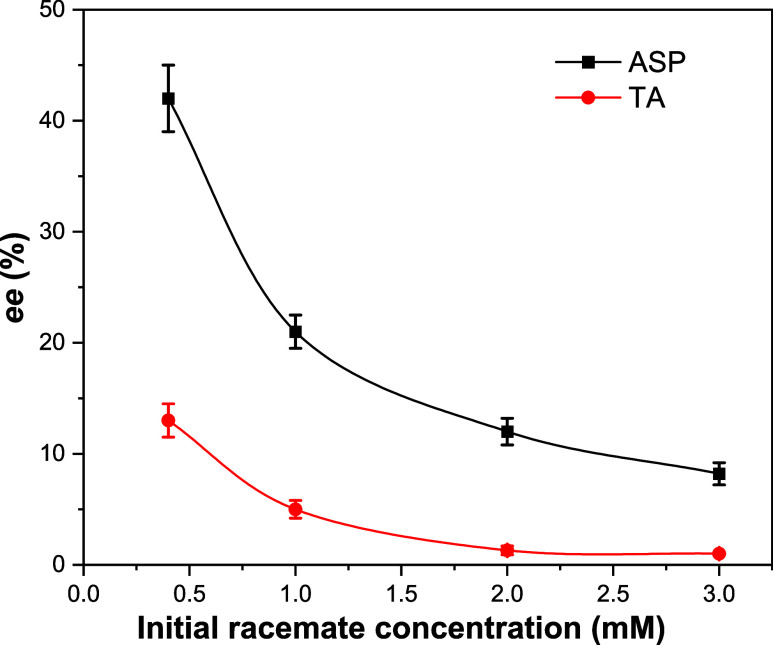
Calculated *ee* for rac-TA and rac-ASP adsorption on enantio-pure NCs. The estimate is based on the measured CD signal of the difference of free TA and ASP enantiomers left in solution after incubation of the NCs with different concentrations of the racemate (indicated on the *x*-axis), divided by the result for the sum of equivalent concentrations of the adsorbed enantiomers taken from the potentiometric titration results. Error bars reflect uncertainties in the estimates of total adsorbed equivalent concentrations from the potentiometric titrations. The lines are a guide to the eye.

Figure 4 shows the results for the *ee* of both TA and ASP racemate adsorption experiments. For rac-ASP, the *ee* climbs to ∼42% at an initial racemate concentration of 0.4 mM and sharply declines to about 8% at 3 mM. The *ee* of the ASP at an initial concentration of 0.2 mM is higher than 50% but was difficult to properly quantify due to relatively noisy CD signal and low total adsorbed *L* + *D*, which was difficult to determine accurately using potentiometric titration. Racemic TA starts with a lower *ee* of 13% at 0.4 mM which decreases to about 1% at 3 mM, revealing a weaker but still noticeable enantioselective interaction. This trend demonstrates that enantiomeric selection in adsorption is higher at lower concentrations for both compounds. A possible reason for this phenomenon would be a reduction in adsorption sites per molecule. While at low concentrations, each molecule would maximize the interactions of its functional groups (carboxyl, hydroxyl, and ammine/ammonium) with surface sites (Tb^3+^, −P–O^–^), at higher concentrations, neighboring sites may be occupied by other molecules, reducing the possibility for enantioselective adsorption. Another plausible factor with increasing influence at high concentrations is the cooperative effects of interactions between the adsorbed molecules at nearest-neighbor sites, of the same or opposite enantiomers. This effect might be somewhat different between TA and ASP, as TA is known to prefer crystallization as a racemate,^26^ i.e l-TA - d-TA interactions are relatively strong, while ASP shows kinetic preference to crystallization as a conglomerate of single enantiomer crystals,^27^ perhaps indicating weaker interenantiomer interactions. This is also implied by the stronger racemate adsorption of TA relative to ASP at the higher racemate concentrations (see initial concentrations of 2.0–3.0 mM, Table S5).

At this point, it is difficult to provide a comprehensive explanation for the difference in adsorption behavior between the two molecules. While the amine group of ASP would probably have higher binding energy to the Tb^3+^ sites compared with the hydroxyl groups of TA, at pH ∼ 3 it might be still protonated and then the relevant adsorption site might be negatively charged phosphate group.^28^ Hence, detailed adsorption simulations are required to further understand the adsorption configurations of the two molecules.

A key finding regarding the surface structure responsible for the molecular adsorption comes from Tb^3+^ and phosphate concentration analysis of the solution which is in contact with the NCs: a comparison of these ionic concentrations in pure water brought in contact with the NCs vs 1 mM ASP solution incubated with the NCs showed that Tb^3+^ ion concentration is low in both cases (15–30 μM). However, phosphate concentration increased substantially on going from pure water (87 μM) to ASP solution (527 μM), indicating that the adsorbing molecules cause the release of surface-bound phosphate groups through stronger coordination to the Tb^3+^ sites. This supports the notion that the adsorption of ASP and probably also TA must be analyzed through the interaction of the functional groups with the Tb^3+^ ions exposed at the surface.

We believe that the presence of two asymmetric carbons in TA vs. a single one in ASP should not be a significant factor, as seen for the adsorption of amino acids on CdS NCs.^29^ Another indication for that comes from previous work which showed that both the closely related malic acid (single asymmetric carbon) and TA have almost equal effect in directing the handedness in the enantioselective preparation of TbPO_4_·H_2_O NCs.^30^

**Figure 5:**
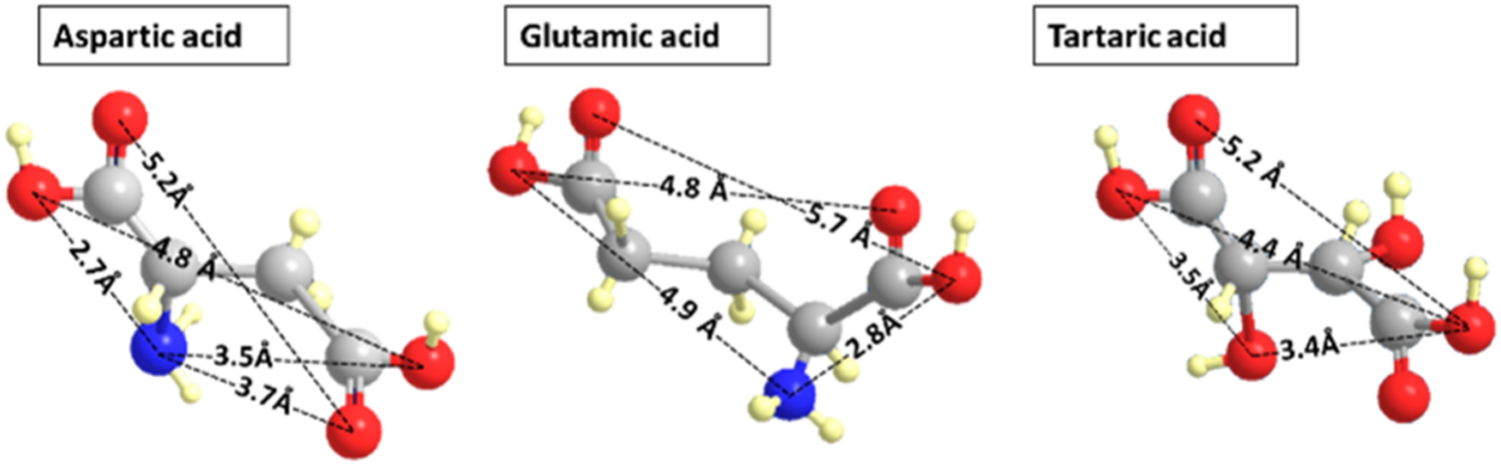
Structure of the three molecules discussed in this work. Note that in glutamic acid a distance close to 4 Å between oxygen atoms or oxygen–nitrogen is absent. Hence, lacking a match to the nearest-neighbor separation of the Tb^3+^ ions.

It should be noted that glutamic acid did not exhibit detectable enantiomeric selectivity in adsorption to the chiral NCs (see Supporting Information, Figure S6). A similar effect was previously observed also for the negligible influence of glutamic acid on the *ee* of the NCs when added to their synthesis instead of TA.^22^ The only difference between ASP and glutamic acid is one additional methylene unit connecting the β-carboxylate group to the asymmetric carbon. Hence, the adsorption enantioselectivity probably arises from specific spatial matching between the ASP functional groups, two carboxylates and one amine, and three Tb^3+^ ion sites at the (100) type surface. Figure 5 shows some interatomic distances for TA, ASP, and glutamic acid, taken from their crystal structures.^31−33^ While in solution or adsorbed to a surface, due to a multitude of possible conformations,^34^ these distances may vary, the distances in Figure 5 provide some rough guide about possible matching between inter-Tb^3+^ distances at the NC surface vs intermolecular oxygen–oxygen or oxygen–nitrogen separations. It can be seen that the nearest-neighbor Tb^3+^ distance is ∼4 Å, matching fairly similar distances (∼3.5–3.7 Å), which occur for oxygen–oxygen and oxygen–nitrogen in TA and ASP, respectively. However, in glutamic acid, a distance close to 4 Å is absent, which indicates that in this case, it may not be possible to form simultaneous binding of the three functional groups to three Tb^3+^ sites. Additionally, there are interoxygen separations in TA and ASP around 5.2 Å, roughly matching the 5.4 Å next-nearest neighbor inter-Tb^3+^ separation. This seems to provide the hypothesized necessary condition for 3-point matching between adsorbed TA and ASP molecules and the TbPO_4_ (100) surface.

Chiral adsorption on NCs was discussed in recent publications both on the TbPO_4_·H_2_O NCs,^30^ and on achiral semiconductor NCs,^35^ where three-point anchoring to the surface was required for a well-defined chiral adsorption configuration, much similar to the minimum of three points required to define a 2D chiral scalene triangle.^36^ This simple criterion, with a simple geometric interpretation, requires specifying at least three interatomic spacings (probably between Tb^3+^ surface sites) to define the handedness of a chiral surface.^37^ Hence, we assume that in ASP such a match occurs between the two carboxyl and amine groups and the Tb^3+^ sites at the surface, while in glutamic acid, one of the carboxyl groups is too far to obtain a good match simultaneously over all three groups. We also tested a case of two anchoring carboxyl groups without a third functional group, in chiral methylsuccinic acid, and indeed no enantioselective adsorption was detected for the racemic mixture adsorbed on either Δ- or Λ-NCs (See Supporting Information, Figure S7).

The single enantiomer adsorption curves could be reasonably fitted with a Langmuir model expression, as seen in Figure 3a,b:
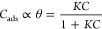
1where θ is the fractional surface coverage, *C*_ads_ is the adsorbed molecule quantity expressed in terms of its equivalent concentration in solution, *K* is the adsorption equilibrium constant, and *C* is the equilibrium molecular concentrations in solution estimated from the CD peak height of the free acid left in solution after separating the adsorbed molecules to the NCs by centrifugation (see details in the Supporting Information). Racemate adsorption could not be modeled by the Langmuir adsorption model (extended for two different adsorbates), probably due to the significant intermolecular interactions, especially at the higher concentrations, which are not addressed by this simple model. These interactions seem to increase adsorption energies and thus bring the racemate adsorption closer to saturation at 5 mM initial concentrations, unlike the single enantiomer adsorption, which seems far from saturation at these conditions, as seen in Figure 3.

The adsorption equilibrium constants of the enantiomers extracted from the Langmuir model fits are presented in Table 1. It can be seen that for both molecules the adsorption constants of l-enantiomer onto Λ-NCs and d-enantiomer onto Δ-NCs were roughly larger by a factor of 2 relative to those of l-enantiomer onto Δ-NCs and d-enantiomer onto Λ-NCs. Also, ASP seems to adsorb stronger than TA (by a factor of ∼2), as might be expected from the observed stronger enantioselectivity in ASP adsorption. As mentioned above, the carboxyl and amino functional groups in ASP apparently facilitate stronger interactions with the chirally arranged Tb^3+^ ions at the surface, compared to the hydroxyl and carboxyl groups of TA.

**Table 1:** Values of Adsorption Equilibrium Constants for the L/d-Enantiomers of TA and ASP Adsorbed on Λ and Δ NCs Extracted from Langmuir Model Fits

**K**	**TA (mM**^–1^**)**	**ASP (mM**^–1^**)**
*K*_*L*_^Λ^	0.10 ± 0.03	0.20 ± 0.04
*K*_*L*_^Δ^	0.05 ± 0.03	0.10 ± 0.01
*K*_*D*_^Δ^	0.10 ± 0.02	0.21 ± 0.04
*K*_*D*_^Λ^	0.04 ± 0.02	0.12 ± 0.02

## Conclusions

High enantioselectivity was demonstrated in a special type of chiral rod-shaped NCs: the TbPO_4_·H_2_O NCs are characterized by predominantly one type of chiral facets, with the highly chiral arrangement of the Tb^3+^ ions, which undergo strong electrostatic/coordinative interactions with at least three functional groups such as carboxyl, amine, and hydroxyl. Even higher enantioselectivity is expected for lower initial ASP concentrations (<0.4 mM). It would be interesting to further explore other multifunctional group chiral (bio)molecules for their enantioselective adsorption on these chiral surfaces. These results are an essential step toward the development of asymmetric heterogeneous catalysis using this type of NCs, which could undergo some surface modifications to enable a wider range of catalytic activity.

## Supporting Information Available

Crystal data, CD vs concentration calibration curves, raw CD data and tables with peak CD values used for adsorbed equivalent concentration determination, potentiometric titration curves used for total adsorbed concentration determination and the corresponding tables with the numerical data analysis, CD data for adsorption of glutamic and methyl succinic acids, and explanation about Langmuir model fits (PDF)
