# Supplementary material for: High Enantioselectivity in Adsorption of Chiral Molecules on the Surface of Chiral Terbium Phosphate Nanocrystals
Source: J Am Chem Soc. 2025 Apr 18;147(17):14191–7. doi: 10.1021/jacs.4c16883 (PMC12046555; doi:10.1021/jacs.4c16883)
Supplement: Supplementary file 1 — ja4c16883_si_001.pdf [file ja4c16883_si_001.pdf]

Supporting Information for:

High enantioselectivity in adsorption of chiral molecules on the surface of chiral terbium phosphate nanocrystals

Abdullah Idrees, Bar Reuven, and Gil Markovich*

School of Chemistry, Tel Aviv University, Tel Aviv 6997801, Israel.

TEM Analysis of $TbPO_4 \cdot H_2O$ NCs

Table S1: The resulting d-spacings (nm) from X-ray diffraction (XRD) and HRTEM analysis along with their corresponding (hkl) planes.

h k l	d-spacing, XRD data (nm)	d-spacing, HRTEM (nm)
(100)	0.594	0.596
(001)	0.687	0.682

Use of CD for analysis of enantioselective adsorption of TA and ASP onto the chiral surfaces of $TbPO_4 \cdot H_2O$ NCs.

The enantioselective adsorption of TA and ASP onto the chiral surfaces of $TbPO_4 \cdot H_2O$ NCs was analyzed using circular dichroism (CD) spectroscopy, following the UV CD peak intensity. Calibration of TA and ASP enantiomer concentrations was done through CD measurements of enantiomerically pure L-TA, D-TA, L-ASP, and D-ASP aqueous solutions at concentrations between 0 - 5 mM, as depicted in Figure S1.

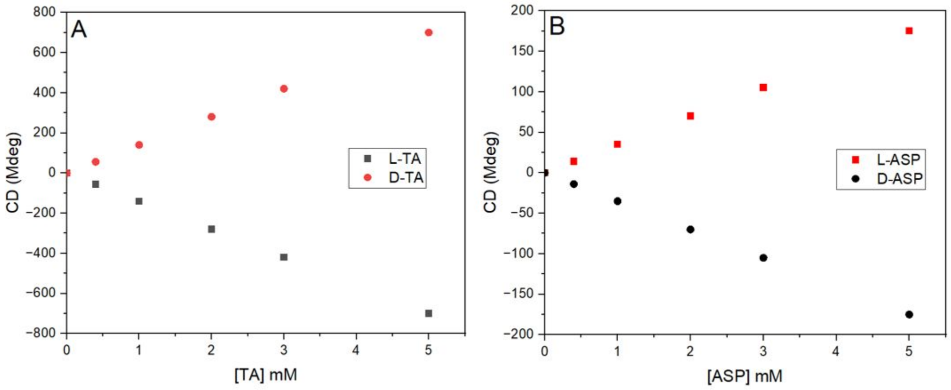

Figure S1. Calibration of TA and ASP CD peak intensities for known enantiomer concentrations: (A) CD peak heights (at 215 nm) of 0 - 5 mM aqueous solutions of L-TA and D-TA. (B) CD peak height (at 204 nm) of 0 - 5 mM aqueous solutions of L-ASP and D-ASP.

Raw CD data after adsorption of single enantiomers of ASP to the NCs

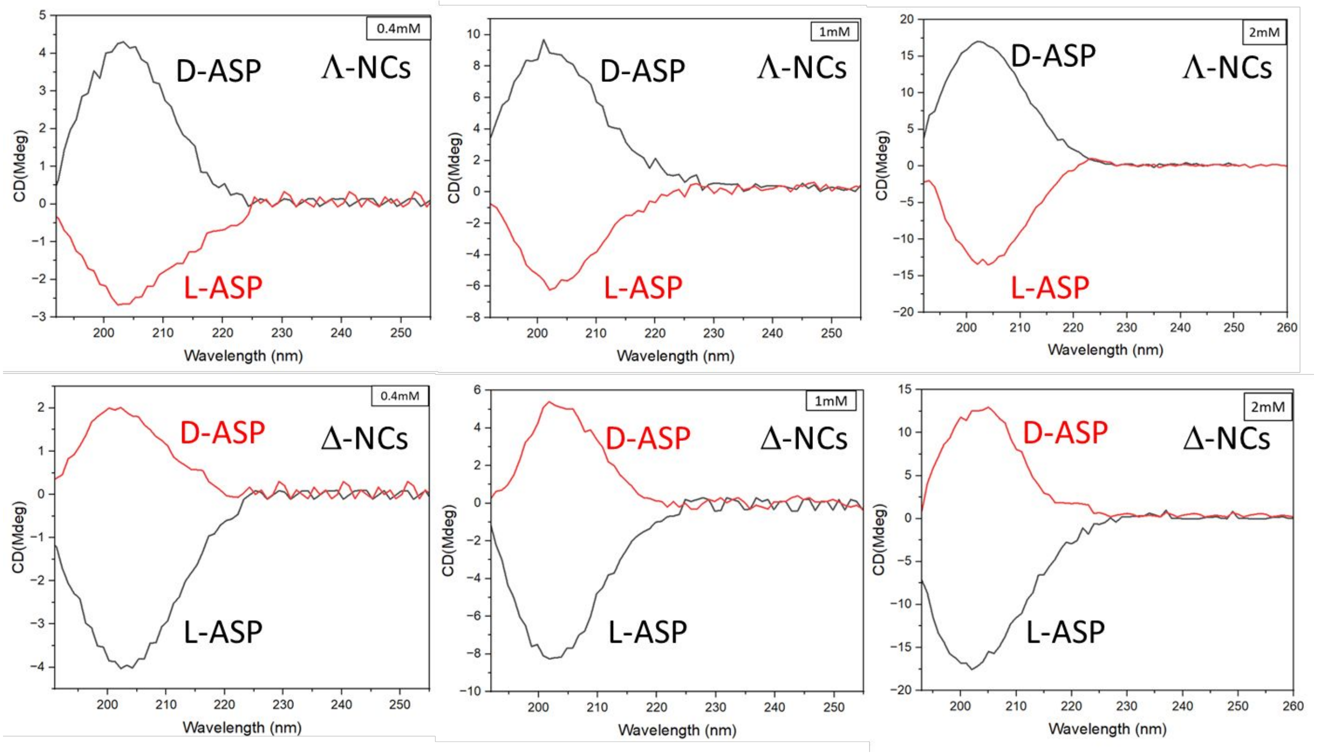

Figure S2. Raw CD spectra of L- and D-ASP solutions from adsorption experiments of L- and D-ASP solutions at different concentrations to either Δ- or Λ-NCs. The measurements are of the free, unadsorbed molecules after removal of the adsorbed molecules and NCs by centrifugation. Hence, the adsorbed equivalent concentration is the difference between the initial (total) concentration indicated at each panel and the free molecules' concentration, determined from the CD calibration curves shown in Fig. S1. The 204 nm peak CD values used for concentration determination were measured after mild smoothing of the curves. These results were used for the plots in Fig. 3b.

Similar results were obtained for TA, but with better signal-to-noise level of the CD spectra, due to larger CD peaks per concentration, as can be seen in Fig. S1.

CD data on free ASP single enantiomer concentrations after adsorption to NCs extracted from CD spectra

Table S2: The CD peak values and estimated free and equivalent adsorbed concentrations of ASP single enantiomers for different initial concentrations.

L/D ASP initial enantiomer concentration (mM)	Free enantiomer CD peak value at 204 nm (mdeg) after adsorption to Λ-NCs	Free enantiomer CD peak value at 204 nm (mdeg) after adsorption to Δ-NCs	Free enantiomer concentration (mM) after adsorption to: Λ-NCs	Δ-NCs	Adsorbed equivalent concentration (mM) to: Λ-NCs	Δ-NCs
L-ASP (0.2)	2.41	1.02	0.07	0.03	0.13	0.17
D-ASP (0.2)	-1.45	-2.10	0.04	0.06	0.16	0.14
L-ASP (0.4)	4.30	2.11	0.12	0.060	0.28	0.34
D-ASP (0.4)	-2.51	-3.80	0.07	0.11	0.33	0.29
L-ASP (1.0)	8.86	5.41	0.25	0.16	0.75	0.84
D-ASP (1.0)	-6.04	-8.38	0.17	0.24	0.83	0.76
L-ASP (2.0)	17.0	12.2	0.49	0.35	1.51	1.65
D-ASP (2.0)	-12.2	-17.0	0.35	0.49	1.65	1.51
L-ASP (3.0)	23.1	17.6	0.66	0.49	2.34	2.51
D-ASP (3.0)	-17.4	-23.3	0.50	0.66	2.50	2.34
L-ASP (5.0)	30.7	26.3	0.88	0.75	4.12	4.25
D-ASP (5.0)	-24.5	-32.4	0.70	0.93	4.30	4.07

CD data on free TA single enantiomer concentrations after adsorption to NCs extracted from CD spectra.

Table S3: The CD peak values and estimated free and equivalent adsorbed concentrations of TA single enantiomers for different initial concentrations

L/D TA initial enantiomer concentration (mM)	Free enantiomer CD peak value at 204 nm (mdeg) after adsorption to Λ-NCs	Free enantiomer CD peak value at 204 nm (mdeg) after adsorption to Δ-NCs	Free enantiomer concentration (mM) after adsorption to: Λ-NCs	Δ-NCs	Adsorbed equivalent concentration (mM) to: Λ-NCs	Δ-NCs
L-TA (0.4)	-14.0	-12.0	0.10	0.09	0.30	0.31
D-TA (0.4)	12.3	13.8	0.09	0.10	0.31	0.30
L-TA (1.0)	-27.3	-25.0	0.20	0.18	0.80	0.82
D-TA (1.0)	22.5	30.0	0.16	0.21	0.84	0.79
L-TA (2.0)	-50	-40	0.36	0.29	1.64	1.71
D-TA (2.0)	40	50	0.29	0.36	1.71	1.64
L-TA (3.0)	-72	-66	0.52	0.47	2.48	2.53
D-TA (3.0)	61	77	0.44	0.55	2.56	2.447
L-TA (5.0)	-111	-101	0.77	0.70	4.23	4.30
D-TA (5.0)	109	105	0.69	0.79	4.31	4.21

Raw CD data after adsorption experiments of racemic mixtures of ASP

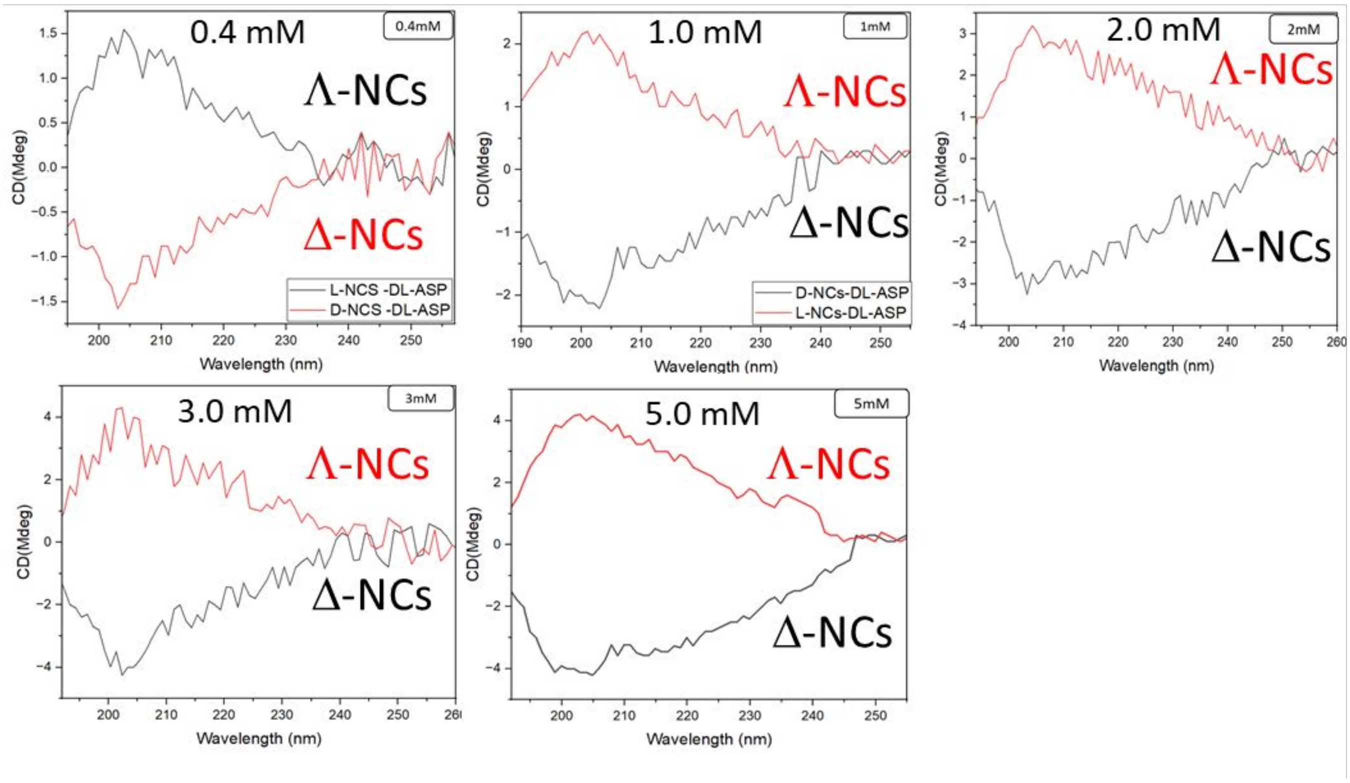

Figure S3. Raw CD spectra, which are the result of adsorption experiments of racemic mixtures of ASP at different initial concentrations to either Δ- or Λ-NCs. The measurements are of the free, unadsorbed molecules after removal of the adsorbed molecules and NCs by centrifugation. The CD peaks reflect the [D-ASP] – [L-ASP] concentration difference. Hence, the adsorbed equivalent concentration difference is [L-ASP] – [D-ASP]. Peak CD values at 204 nm were taken after mild smoothing of the curves

Table S4: CD and concentration difference data from rac-ASP adsorption to the NCs.

Initial rac-ASP concentration (mM)	CD peak of unadsorbed molecules after adsorption to Δ-NCs (mdeg)	CD peak of unadsorbed molecules after adsorption to Λ-NCs (mdeg)	Enantiomer concentration difference averaged over Λ- and Δ-NCs (mM)
0.4	-1.38	1.39	0.040
1.0	-2.08	2.10	0.060
2.0	-2.90	3.02	0.085
3.0	-3.91	3.97	0.113
5.0	-4.08	4.10	0.117

Raw CD data after adsorption experiments of racemic mixtures of TA

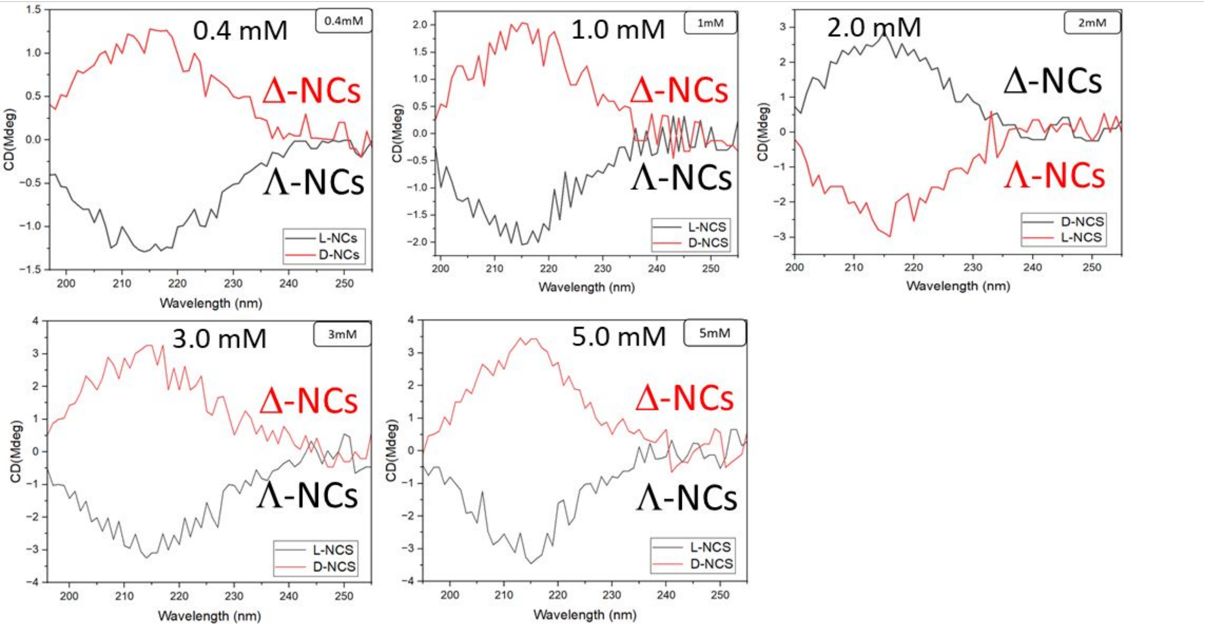

Figure S4. Raw CD spectra, which are the result of adsorption experiments of racemic mixtures of TA at different concentrations to either Δ- or Λ-NCs. The measurements are of the free, unadsorbed molecules after removal of the adsorbed molecules and NCs by centrifugation. The CD peaks reflect the [D-TA] – [L-TA] concentration difference. Hence, the adsorbed equivalent concentration difference is [L-TA] – [D-TA]. Peak CD values at 215 nm were taken after mild smoothing of the curves.

Table S5: CD and concentration difference data from rac-TA adsorption on the NCs.

Initial rac-TA Concentration (mM)	CD peak of unadsorbed molecules after adsorption to Δ-NCs (mdeg)	CD peak of unadsorbed molecules after adsorption to Λ-NCs (mdeg)	Enantiomer concentration difference averaged over Λ- and Δ-NCs (mM)
0.4	-1.3	1.2	0.009
1.0	-1.9	1.9	0.014
2.0	-2.6	2.6	0.019
3.0	-3.0	3.1	0.022
5.0	-3.1	3.3	0.023

Potentiometric titration curves to determine the equivalent adsorbed concentration of L+D molecules in racemate adsorption experiments

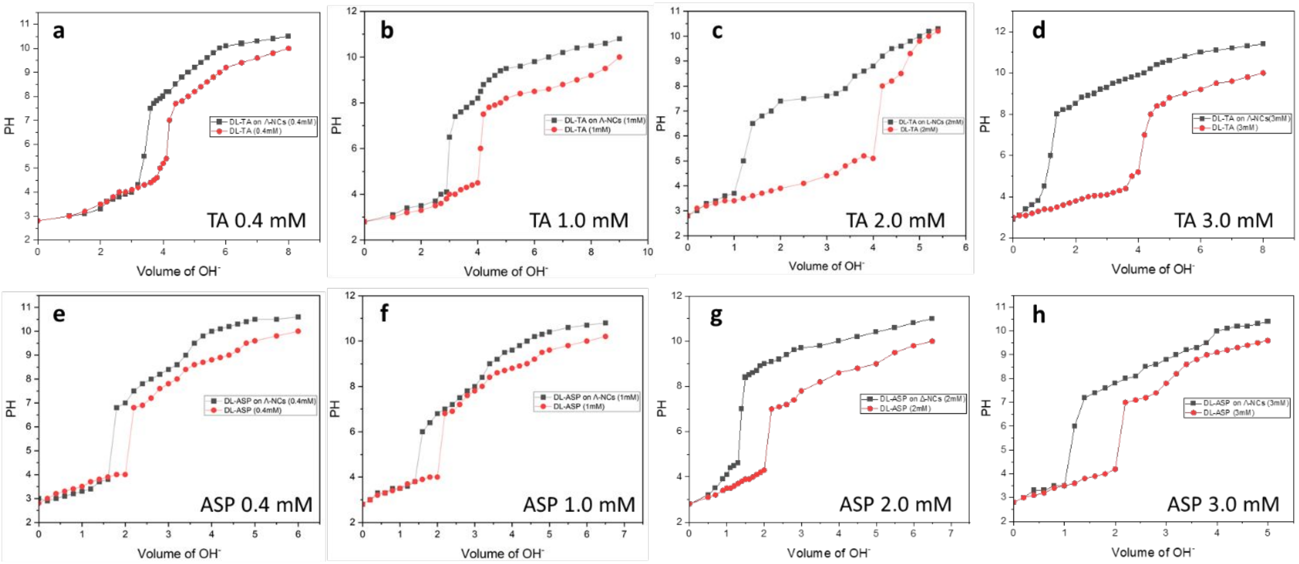

Figure S5. (a-d) Potentiometric titration curves for determining the free [L-TA] + [D-TA] concentrations for different initial racemate concentrations. 2 mL of solutions of rac-TA were the control sample (red) and of free (unadsorbed) L+D TA molecules after adsorption from the racemate solution onto Λ-NCs and separation from the NCs (black), titrated with NaOH solutions of the same initial concentration. (e-h) Potentiometric titration curves for determining the free L+D ASP concentrations. 2 mL of solutions of rac-ASP were the control samples (red) and of free (unadsorbed) L+D ASP molecules after adsorption from the racemate solution onto Λ-NCs and separation from the NCs (black), titrated with NaOH solutions of the same initial concentration.

Note, that in the case of TA, 4 mL of the base were needed to titrate the reference samples, since the TA was initially in its doubly protonated state, and a double amount of base was needed to remove both protons, where only the removal of the second proton can be clearly seen due to small separation of the two pk_a values. In ASP solutions, one of the carboxylates is already initially deprotonated, hence only half of the base quantity was required to remove the second acidic proton (the amine's proton is removed at higher pH).

The equivalence points were determined by taking the peak of the derivative of the titration curves.

The titration curve of the control sample was used as a reference and the equivalence points of the titrations of the actual free acid concentration after adsorption were determined relative to the control sample, see details in the Experimental Section of the paper.

Summary of potentiometric titration results

The data presented in Table S6 describe the adsorption behavior of ASP and TA racemic mixtures at various initial concentrations on the NCs. The table compares the initial concentrations of each acid before the adsorption experiment with the corresponding adsorbed equivalent and free acid concentrations.

For both acids, the adsorbed equivalent concentration increases significantly with the increase in the initial (total) concentration. In the case of rac-ASP it ranges from 0.074 mM at an initial concentration of 0.4 mM to 1.38 mM at an initial concentration of 3.0 mM. Similarly, for TA, the adsorbed equivalent concentration goes from 0.067 mM at an initial concentration of 0.4 mM up to 2.14 mM at an initial concentration of 3.0 mM. The adsorbed equivalent acid concentrations continue to increase with the rise in total concentration, suggesting that surface saturation is not yet achieved at this concentration range (as in the case of single enantiomer adsorption). The relative increase in adsorbed vs. free acid concentrations might indicate stabilizing inter-molecular (also between opposite enantiomers) interactions at the surface of the NCs, which increase adsorption energy at high surface coverage.

Table S6: Concentration analysis of adsorption experiments of racemic mixtures of ASP and TA on the NCs: Total (initial) racemate concentration, free (unadsorbed) L+D concentration determined by titration, adsorbed equivalent concentration of [L]+[D] = initial – free [L]+[D], and estimated adsorbed $ee = {|[L]-[D]|_{ads}}/{([L]+[D])_{ads}}$.

**[L-ASP] + [D-ASP] (mM)**				
Total concentration (mM)	0.4	1.0	2.0	3.0
Free acid concentration (mM)	0.326	0.715	1.30	1.62
Adsorbed equivalent concentration (mM)	0.074	0.285	0.70	1.38
ee using data from Table S4 (%)	**42**	**21**	**12**	**8.2**
**[L-TA] + [D-TA] (mM)**				
Total concentration (mM)	0.4	1.0	2.0	3.0
Free acid concentration (mM)	0.333	0.72	0.59	0.86
Adsorbed equivalent concentration (mM)	0.067	0.28	1.41	2.14
ee using data from Table S5 (%)	**13**	**5**	**1.3**	**1.0**

Glutamic acid adsorption on the $TbPO_4 \cdot H_2O$ NCs

Glutamic acid (chemical structure shown in Figure S6) did not exhibit any measurable enantiomeric selectivity during its adsorption onto the chiral nanocrystals NCs. Despite the inherent chirality of the NC surfaces, no significant enantiomeric difference in the adsorption behavior of the 1 mM racemic glutamic acid was observed.

Figure S6 shows the CD spectra of 1 mM racemic glutamic acid, where no CD signal is observed after adsorption to the chiral NCs. The absence of a significant CD signal indicates the lack of enantiomeric selectivity in its adsorption.

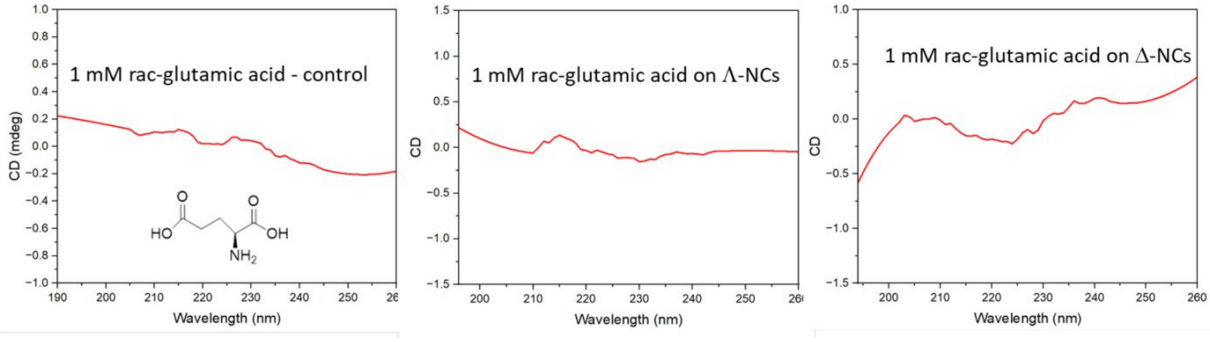

Figure S6. CD spectra of 1 mM racemic glutamic acid before (left), and after being in contact with Λ-NCs and Δ-NCs and separation of the supernatant. The signal is practically zero, within the noise level. Hence, no significant enantioselectivity is observed for glutamic acid adsorption. The structure of the glutamic acid molecule is also shown.

Methyl succinic acid adsorption on the NCs

Methyl succinic acid (chemical structure shown in Figure S7) did not display any detectable enantiomeric selectivity during its adsorption onto the chiral NCs.

Figure S7 shows the absence of any detectable CD signal after adsorption of the racemate of methyl succinic acid to the chiral NCs, hence it does not exhibit enantioselective adsorption to the chiral NCs.

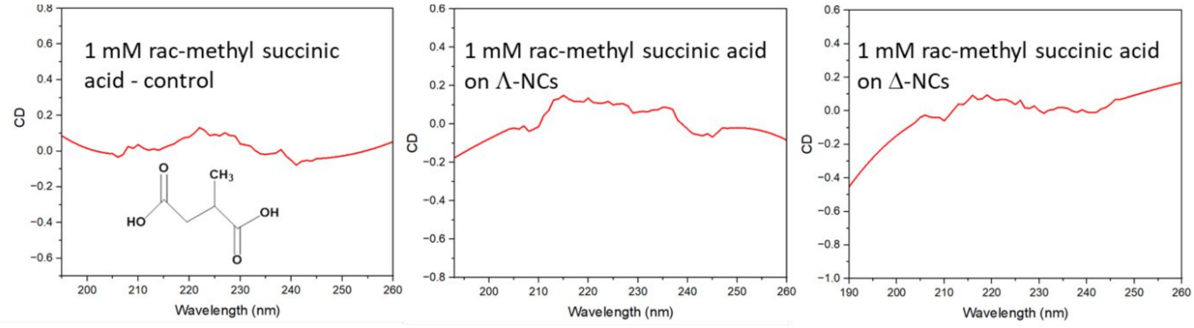

Figure S7. CD spectra of 1 mM racemic methyl-succinic acid before (left) and after being in contact with Λ-NCs and Δ-NCs and separation of the supernatant. The signal is practically zero, within the noise level. Hence, no significant enantioselectivity is observed for methyl-succinic acid adsorption. The structure of the methyl-succinic acid molecule is also shown.

Langmuir model fits

The Langmuir model assumes monolayer adsorption onto the surface containing a finite number of identical adsorption sites without any lateral interactions between the adsorbed molecules.

For the adsorption of L-TA, D-TA, L-ASP, and D-ASP onto chiral $TbPO_4 \cdot H_2O$ NC surfaces, the fraction of occupied sites can be expressed in terms of the equivalent concentration of the adsorbed molecules. The Langmuir adsorption isotherm equation relates the concentration of adsorbed species to the equilibrium concentration of the free species in the solution . We can write the Langmuir equation as follows:

$$C_{ads} \propto \theta = \frac{KC}{1 + KC}$$

- θ is the fractional surface coverages of either L-TA, L-ASP, D-TA, or D-ASP adsorbed either on Δ-NCs or Λ-NCs.
- C_{ads} is the adsorbed equivalent concentration of L-TA. D-TA. L-ASP or D-ASP onto Δ-NCs or Λ-NCs.
- K is adsorption equilibrium constant for L-TA, D-TA, L-ASP, or D-ASP adsorbed on Δ-NCs or Λ-NCs.
- C is the equilibrium molecular concentration in solution estimated from the CD peak height of free acid left in solution after removing the molecules adsorbed to the NCs by centrifugation
